# Blocking AREG-EGFR signaling attenuates pan-arterial fibrosis in chronic cardiac allograft rejection

**DOI:** 10.7150/thno.125318

**Published:** 2026-02-11

**Authors:** Kai Xing, Yuan Chang, Yuqi An, Xiao Chen, Xiaofei Zhu, Jian Huang, Peiyuan Li, Mengda Xu, Yixuan Sheng, Xingchao Du, Hao Liu, Jiangping Song

**Affiliations:** 1 Department of Cardiac Surgery, Fuwai Hospital, National Center for Cardiovascular Diseases, Chinese Academy of Medical Sciences and Peking Union Medical College, Beijing, China.; 2 State Key Laboratory of Cardiovascular Disease, Fuwai Hospital, National Center for Cardiovascular Diseases, Chinese Academy of Medical Sciences and Peking Union Medical College, Beijing, China.; 3 Beijing Key Laboratory of Organ Xenotransplantation, Fuwai Hospital, National Center for Cardiovascular Diseases, Chinese Academy of Medical Sciences and Peking Union Medical College, Beijing, China.; 4 First Teaching Hospital of Tianjin University of Traditional Chinese Medicine, Tianjin, China.; 5 Department of Urology Surgery, Beijing Jishuitan Hospital, Beijing, China.; 6 Department of Cardiac Surgery, Fuwai Yunnan Hospital, Chinese Academy of Medical Sciences, Affiliated Cardiovascular Hospital of Kunming Medical University, Kunming, China.; 7 Shenzhen Key Laboratory of Cardiovascular Disease, Fuwai Hospital, Chinese Academy of Medical Sciences, Shenzhen, China.; 8 Sanya Institute of China Agricultural University, Sanya, China.

**Keywords:** heart transplantation, pan-arterial fibrosis, cardiac allograft vasculopathy, AREG-EGFR

## Abstract

**Background:**

Cardiac allograft vasculopathy (CAV) is a major barrier to long-term survival after heart transplantation, characterized by progressive vascular remodeling and luminal narrowing. Fibrosis is one of the key pathological features of CAV progression, but its underlying mechanisms remain unclear. This study aims to investigate the mechanisms of CAV-associated vascular fibrosis and explore potential therapeutic targets.

**Methods:**

Clinical specimens from the aorta (AO), pulmonary artery (PA), and coronary artery (CA) of CAV and control (Ctrl) groups were analyzed using single-cell RNA sequencing (scRNA-seq). Further validation was performed using a mouse arterial transplantation model.

**Results:**

This study found that vascular fibrosis occurs extensively in AO, PA, and CA, rather than being confined to CA alone. scRNA-seq analysis revealed that increased fibroblasts (FBs) and extracellular matrix (ECM) remodeling are common features across all three vascular regions. Cell-cell interaction analysis showed that T cells promote FB activation via AREG-EGFR. Two murine transplantation models further confirmed that blocking AREG-EGFR signaling significantly reduces fibrosis.

**Conclusion:**

Pan-arterial fibrosis represents a unifying pathological process across major vascular territories in CAV. Targeting fibrotic remodeling may offer a promising adjunctive strategy to improve long-term graft outcomes.

## Introduction

Cardiac allograft vasculopathy (CAV) is the primary barrier to long-term survival following heart transplantation (HTx), characterized by progressive vascular remodeling, including concentric intimal thickening and luminal narrowing 1, 2. Approximately 25% of transplant recipients develop CAV within five years post-transplantation, with the incidence rising to nearly 50% within a decade, making it a leading cause of graft failure 3-5. The vascular changes observed in cardiac allografts result from a prolonged pathological process driven by chronic rejection, which is triggered by the recipient's immune response to donor allo-antigens 1, 4, 6-8. The persistent activation of immune cells leads to the release of cytokine signaling, which stimulates aberrant proliferation of fibroblasts (FBs) and smooth muscle cells (SMCs), promoting extracellular matrix (ECM) deposition, vascular wall thickening, and ultimately, fibrosis 1, 2, 9.

This extensive vascular remodeling not only affects the coronary arteries (CA) but also involves the major vessels of the transplanted heart, including the aorta (AO) and pulmonary artery (PA). In these large vessels, luminal narrowing is typically less pronounced due to their larger diameter; however, structural alterations in the vessel walls lead to increased vascular stiffness and impaired diastolic function. A study of 39 pediatric HTx recipients reported that, after a median follow-up of three years, the aortic distensibility index decreased by approximately 50%, while the stiffness index doubled 10. Additionally, another study identified aortic pulse wave velocity, a key indicator of aortic stiffness, as an independent risk factor for progressive CAV 11. Similarly, PA pressure, which initially declines within the first three years post-HTx, has been observed to rise again between the third and fifth years, correlating with poorer prognosis 12. These findings suggest that CAV affects both the CA and major arteries, contributing to diffuse vascular dysfunction and ultimately leading to graft failure.

Although immunosuppressive therapy has significantly slowed the progression of CAV, its onset and development remain irreversible. This study systematically analyzed the pathological changes in AO, PA, and CA of CAV patients. The results revealed common pathological features among these arteries, particularly severe vascular fibrosis driven by FB activation. Further single-cell RNA sequencing (scRNA-seq) demonstrated that FB activation leads to extensive ECM remodeling. Cellular interaction analysis revealed a key mechanism by which T cells secrete amphiregulin (AREG) to drive vascular remodeling. Animal experiments further confirmed that blocking the AREG-EGFR signaling pathway effectively attenuates fibrosis in the vascular walls of allografts. In summary, this study defines pan-arterial fibrosis as a unifying feature across coronary, pulmonary, and aortic vessels in CAV, offering new perspectives on its pathogenesis and treatment.

## Methods

### Human specimen collection

This study strictly adhered to the ethical principles outlined in the Declaration of Helsinki and was approved by the Ethics Committee of Fuwai Hospital, Chinese Academy of Medical Sciences (Approval No.: 2013-049). Written informed consent was obtained from all patients or their legal representatives after a detailed explanation of the study objectives and the intended use of human tissue samples. A total of six specimens were included in this study, comprising three from CAV patients (one subjected to scRNA-seq, **Table 1**) and three from non-diseased controls. Control (Ctrl) specimens were obtained from donor hearts that could not be transplanted due to size mismatch, while CAV specimens were collected from recipients undergoing re-HTx due to severe disease progression. The AO and PA specimens were obtained from the donor heart, not recipient vessels, while the CA specimens were collected from the left anterior descending branch. All specimens were provided by Fuwai Hospital and were processed under stringent collection and preservation protocols to ensure sample integrity and the reliability of research outcomes.

### Single-cell RNA sequencing (scRNA-seq)

#### Cardiac cell isolation and flow cytometry sorting

Tissue samples from the aorta (AO), pulmonary artery (PA), and coronary artery (CA) were immediately placed in ice-cold DMEM (Gibco, 11885084). After PBS washing, tissues were minced and digested in HBSS containing 600 IU/mL collagenase II (Worthington, 43J14367B) at 37 °C for 15 min. The suspension was filtered through a 70 μm strainer, enzymatic activity was neutralized with an equal volume of 10% FBS/DMEM, and cells were centrifuged (400 × g, 5 min, 4 °C) and washed 3-5 times, then resuspended in 2% FBS/DMEM. Red blood cells were removed with RBC lysis buffer (Beyotime, C3702) for 10 min, followed by filtration and resuspension in ice-cold 2% FBS/DMEM. Cells were stained with 7-AAD (BD, 559925; 1:20) and 7-AAD⁻ viable cells were sorted on a FACS Aria II (BD) for scRNA-seq.

#### Library construction, sequencing, and data processing

Sorted cells were processed on the 10x Genomics Chromium platform to generate GEMs using the Single Cell 5′ Library & Gel Bead Kit (10×, 1000006) and Chromium Single Cell A Chip Kit (10×, 120236). Reverse transcription was performed (53 °C 45 min; 85 °C 5 min; hold 4 °C). cDNA was purified (DynaBeads MyOne Silane) and QC'd (Agilent Bioanalyzer High Sensitivity). Libraries were sequenced on an Illumina HiSeq X-ten (150-cycle high output). Raw data were processed with Cell Ranger 6.1.2 and aligned to the human reference genome (GRCh38/hg38) to generate UMI count matrices.

#### Sample integration, dimensionality reduction, and clustering (BBKNN)

Count matrices were imported into Scanpy (AnnData). Standard QC filters were applied (cells with < 500 or > 11,000 UMIs, mitochondrial gene fraction > 10%, or ribosomal gene fraction > 20% were removed). Data were normalized and log-transformed; highly variable genes were selected, followed by PCA. Batch correction / integration was performed using BBKNN (batch_key specifying sample/origin). Neighborhood graphs were computed on the BBKNN graph, UMAP was used for visualization, and clustering was performed with the Leiden algorithm (resolution = 0.4). Differentially expressed genes were identified and cell types annotated based on canonical markers. Endothelial subclustering used Leiden (resolution 0.1-1.0) with low-quality/heterogeneous clusters excluded.

#### Gene ontology (GO) enrichment and cell-cell communication analysis

GO enrichment was performed with clusterProfiler (enrichGO; org.Hs.eg.db as background). Cell-cell communication was inferred with CellChat using the built-in CellChatDB.human ligand-receptor database. Default workflow settings were applied for probability estimation and pathway-level aggregation; network plots were generated with the package's functions.

### Histological staining

Cardiac tissues were collected, rinsed with PBS to remove blood, and fixed in 10% neutral buffered formalin for 24-48 hours. The samples were then subjected to a gradient dehydration process (50% to absolute ethanol), followed by xylene clearing and paraffin embedding at 60 °C. Paraffin blocks were sectioned at a thickness of 4 μm, spread in a 40 °C water bath, transferred onto glass slides, and dried for further analysis.

For hematoxylin and eosin (H&E) staining, sections were baked at 65 °C for 2 hours, deparaffinized, rehydrated, and sequentially stained with hematoxylin and eosin for 5 minutes each, with intermittent washing under running water. The stained sections were then dehydrated, cleared, and mounted with neutral resin for microscopic observation.

Masson's trichrome staining was performed using a Masson staining kit (Solarbio, G1340). The deparaffinization and rehydration steps were identical to those used in H&E staining. Sections were sequentially stained with Weigert's iron hematoxylin, Ponceau acid fuchsin, phosphomolybdic acid, and aniline blue, with intermittent washing using weak acid working solution to remove excess stain. Finally, the sections were dehydrated, cleared, and mounted for microscopic examination.

Elastic Van Gieson (EVG) staining was performed using an EVG staining kit (Solarbio, G1597). The deparaffinization and rehydration procedures were identical to those used for H&E staining. Sections were first incubated with modified VG solution for 10 minutes, followed by a brief rinse with distilled water (5-20 seconds) to remove excess stain. They were then stained with Verhoeff staining solution for 5 minutes and rinsed again with distilled water (5-10 seconds). Differentiation was carried out using Verhoeff differentiator for 5-10 seconds until elastic fibers became clearly visible, followed by another brief rinse. Finally, the sections were dehydrated through graded ethanol, cleared in xylene, and mounted with neutral resin for microscopic observation.

All sections were imaged under a light microscope to capture representative fields. Quantitative analysis was performed using ImageJ software. The average thickness of the intima and media was measured in each vascular cross-section to calculate the intima-to-media thickness ratio (I/M ratio). The collagen volume fraction (CVF) was determined from Masson-stained images as the percentage of blue-stained collagen fibers relative to the total vessel wall area. Data are presented as mean ± standard deviation (SD).

### Multiplex immunohistochemistry (mIHC)

Multiplex immunohistochemistry (mIHC) was performed using the Opal 7-Color Manual IHC Kit (Akoya Biosciences, NEL811001KT). Tissue sections underwent the same deparaffinization and rehydration procedures as described for H&E staining. After antigen retrieval and blocking with goat serum, sections were incubated overnight at 4 °C in a humidified chamber with primary antibodies against AREG (Santa Cruz Biotechnology, sc-74501), CD3 (Proteintech, 17617-1-AP), vimentin (Abcam, ab8978), PDGFRα (Abcam, ab96569), and α-SMA (Abcam, ab184705). Following primary antibody incubation, sections were treated with an HRP-conjugated secondary antibody and developed with the corresponding Opal fluorophore via tyramide signal amplification. Antibody stripping was performed by heating in retrieval buffer before the next staining cycle. After completing all labeling steps, nuclei were counterstained with DAPI (ThermoFisher, P36971), and sections were mounted with antifade mounting medium for imaging.

### Mouse artery and heart transplantation model

Male BALB/c (H-2d) and C57BL/6 (H-2b) mice, aged 6-8 weeks, were obtained from Beijing Vital River Laboratory Animal Technology Co., Ltd. Bm12 (H2-Ab1^bm12^) mice were purchased from The Jackson Laboratory. Prior to the experiment, all mice were housed in a specific pathogen-free (SPF) environment with ad libitum access to food and water. All procedures strictly adhered to the Guide for the Care and Use of Laboratory Animals and were approved by the Institutional Animal Care and Use Committee of Fuwai Hospital (Approval No.: 0108-7-300-ZX(X)-053).

In this study, C57BL/6 mice were used as recipients, while BALB/c mice served as donors. The transplantation model was established according to previously described methods 13-17. Briefly, donor BALB/c mice were deeply anesthetized and underwent median sternotomy. The thoracic aorta between the diaphragm and the aortic arch was dissected, branch vessels were ligated sequentially, and a 5-8 mm segment was excised. The lumen was gently flushed with cold heparinized normal saline, and the segment was kept in pre-chilled sterile saline with cold ischemic time minimized. A polytetrafluoroethylene micro-cuff matching the internal diameter of the recipient common carotid artery was selected. Recipient mice were anesthetized and placed supine. After sterile preparation, the common carotid artery was exposed and mobilized; proximal and distal microvascular clamps were applied, the artery was transected, and both ends were flushed with heparinized saline. The proximal and distal arterial stumps were sleeved onto the prepared cuff with the vessel wall everted over the cuff shoulder and secured circumferentially in the cuff groove with 8-0 silk to ensure a patent, tear-free lumen. The donor aortic segment was then sleeved over the outer surfaces between the two cuffed stumps, alignment was adjusted to avoid torsion or undue tension, and each interface was tied circumferentially with 8-0 silk, confirming smooth edges without folds or leakage. Reperfusion was achieved by slowly releasing the distal and then the proximal clamp, graft filling and color change were observed, and the incision was closed in layers. Postoperatively, mice recovered in a temperature-controlled chamber with analgesia and antibiotic prophylaxis as needed.

For heart transplantation, a well-established chronic cardiac transplantation model was used. C57BL/6 mice served as recipients, and bm12 mice were used as donors to establish a cervical heterotopic heart transplantation model 18-20. Briefly, recipient mice were placed in the supine position under general anesthesia, and a midline cervical incision was made to expose and isolate the common carotid artery and external jugular vein. The distal ends of the vessels were ligated, the proximal ends were transected, and the vessel stumps were sleeved onto microvascular cuffs matched to their internal diameters and secured with 8-0 silk sutures. Donor bm12 mice were deeply anesthetized and subjected to thoracotomy; the heart was rapidly excised after perfusion with approximately 1 mL of normal saline via the inferior vena cava, and the ascending aorta and pulmonary artery were trimmed to appropriate lengths. The donor aorta and pulmonary artery were then sleeved over the outer surfaces of the cuffed recipient common carotid artery and external jugular vein, respectively, and circumferentially secured with 8-0 silk sutures, taking care to avoid torsion or excessive tension. Blood flow was restored by sequentially releasing the vascular clamps, and successful reperfusion was confirmed by a change in graft color to pink and the resumption of spontaneous beating. The incision was closed in layers, and mice were allowed to recover in a temperature-controlled environment with analgesia and antibiotic prophylaxis provided as needed.

Mice were randomly assigned to the treatment and control groups. The treatment group received Erlotinib (HY-50896, MCE) at a dose of 50 mg/kg/day by oral gavage. The control group received an equal volume of vehicle on the same schedule. At the end of the treatment period, all mice were euthanized, and tissues were collected for further analysis.

### Statistical analysis and data visualization

All experimental data were expressed as mean ± standard deviation (mean ± SD). Differences between two groups were analyzed using Student's t-test. All statistical analyses and data visualization were conducted using GraphPad Prism 8, with statistical significance set at *p* < 0.05.

## Results

### Pan-arterial pathological features of arterial remodeling in CAV

**Figure 1:**
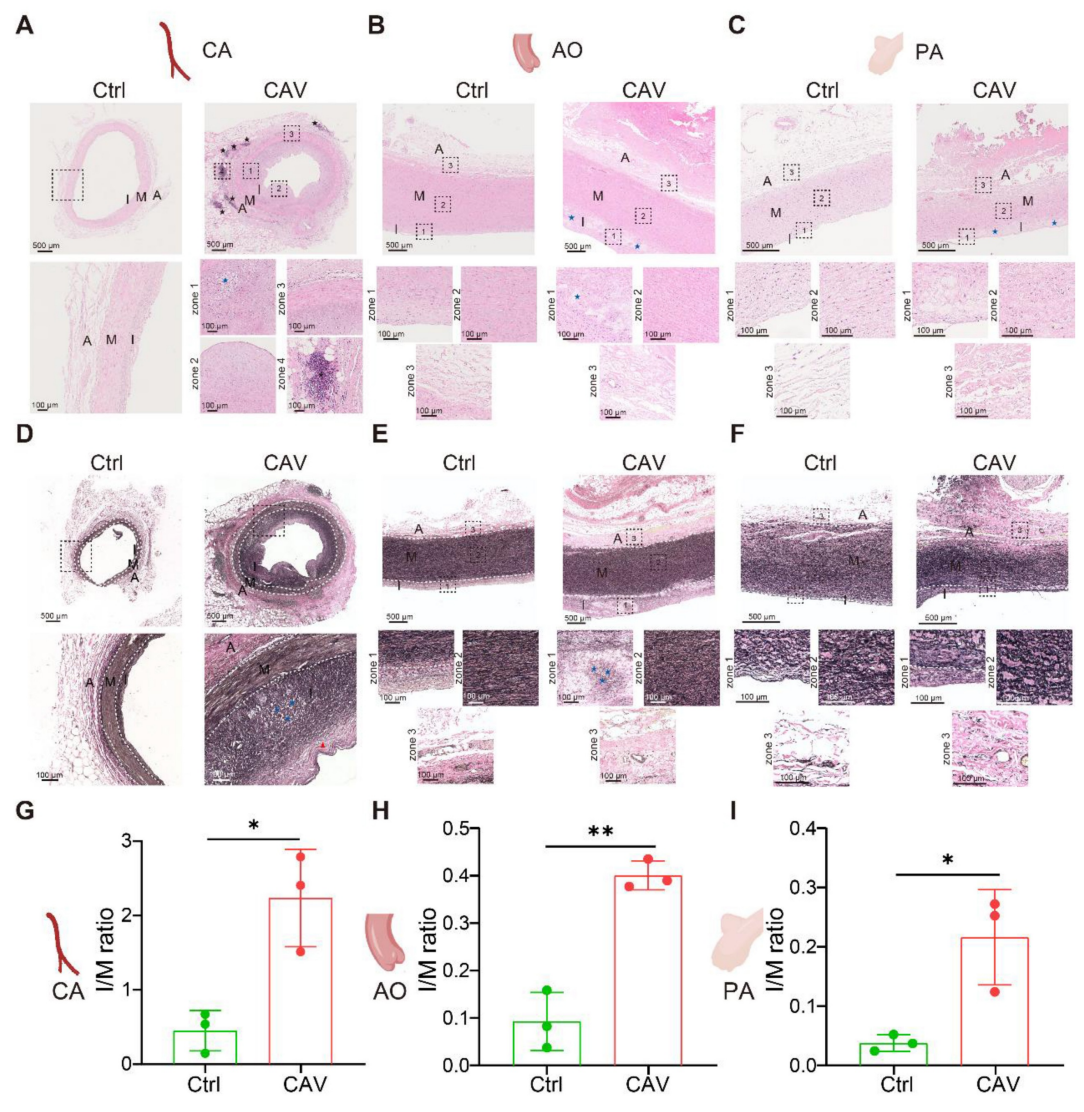
** H&E and EVG staining reveal consistent features of arterial remodeling in coronary, aortic, and pulmonary vessels from CAV allografts.** (A) In the CAV group, coronary arteries (CA) exhibit marked intimal hyperplasia, luminal narrowing, foam cell accumulation, and lipid deposition, with prominent lymphoid cell aggregates observed in the adventitia; in contrast, control arteries show preserved architecture with only mild adaptive intimal thickening. (B) Aortas (AO) from the CAV group display significant intimal thickening and plaque-like structures, while control vessels remain structurally intact. (C) Pulmonary arteries (PA) in the CAV group show similar lesions, characterized by intimal expansion and foam cell infiltration, which are absent in controls. (D) EVG staining reveals pronounced intimal thickening in CAV coronary arteries, accompanied by collagen accumulation, foam cell infiltration, and disrupted elastic fibers in the media; control arteries exhibit continuous and well-organized elastic fibers. (E) CAV aortas display intimal thickening and elastic fiber disorganization, whereas control aortas show normal architecture. (F) CAV pulmonary arteries demonstrate remodeling patterns similar to those observed in coronary and aortic vessels, while control arteries maintain intact structure. (G-I) The intima-to-media (I/M) thickness ratio is significantly increased in CAV CAs, AOs, and PAs, indicating widespread intimal remodeling. A: adventitia; M: media; I: intima. Blue asterisks indicate foam cell-rich regions; black asterisks indicate lymphoid cell aggregates; Red arrows indicate thick fibrous caps. Data are presented as mean ± SD (n = 3). *p < 0.05, **p < 0.01 (Student's t-test).

To investigate structural alterations across different vascular beds during CAV, we performed H&E staining on coronary arteries (CA), aorta (AO), and pulmonary arteries (PA) from allograft specimens and compared them with non-transplanted healthy controls. In CAV coronary arteries, there was marked intimal thickening leading to severe luminal narrowing. The neointima was predominantly composed of smooth muscle-like cells, indicative of a muscular-type intimal remodeling pattern. In addition to structural narrowing, characteristic atherosclerotic changes were observed, including abundant foam cells and extensive intra- and extracellular lipid deposition, suggesting an atherogenic process associated with dysregulated lipid metabolism in CAV. In contrast, control coronary arteries exhibited only mild adaptive intimal thickening with preserved architecture and no evidence of inflammation or lipid accumulation (**Figure 1A**).

Another notable feature of CAV coronary arteries was the presence of lymphoid cell aggregates in the adventitia, characterized by dense clusters of lymphocytes. Previous studies have reported that the formation of tertiary lymphoid organ (TLO)-like structures is associated with CAV progression, and that localized immune activation within these aggregates may contribute to graft vascular fibrosis and occlusion 17, 21-25. In our samples, these lymphoid aggregates predominantly consisted of mixed lymphocyte populations, suggesting an early or less organized stage of lymphoid structure formation rather than fully mature TLOs 24-26. In addition, widespread immune cell infiltration was observed across the intima, media, and adventitia, indicating that CAV involves multi-layered immune-mediated vascular remodeling.

Similar pathological alterations were observed in the aorta and pulmonary artery of allografts (**Figure 1B-C**). Compared with controls, both vessels exhibited substantial intimal thickening with the formation of atherosclerotic-like plaques and the presence of foam cells. These consistent changes across vascular territories suggest that CAV affects not only coronary arteries but also extends to large vessels, highlighting a pan-arterial pathological process.

To further characterize vascular remodeling during CAV, EVG staining was performed on CA, AO, and PA to assess collagen deposition and elastic fiber integrity. In CAV CAs, there was marked intimal thickening with extensive collagen accumulation in the intima and adventitia, accompanied by atherosclerotic plaques and foam cell infiltration (**Figure 1D**). Elastic fibers in the media appeared fragmented and disorganized, indicating structural disruption. In contrast, control vessels showed preserved architecture with continuous elastic fibers and minimal collagen. Similar changes were observed in AO and PA from CAV allografts, including intimal expansion, elastic fiber disarray, and accumulation of collagen and foam cells (**Figure 1E-F**), consistent with the pattern in CA. To quantify intimal remodeling, the intima-to-media (I/M) thickness ratio was calculated. CAV vessels showed significantly higher I/M ratios across all three vascular beds compared to controls (**Figure 1G**-**I**), indicating widespread intimal hyperplasia and vascular structural abnormalities in CAV.

**Figure 2:**
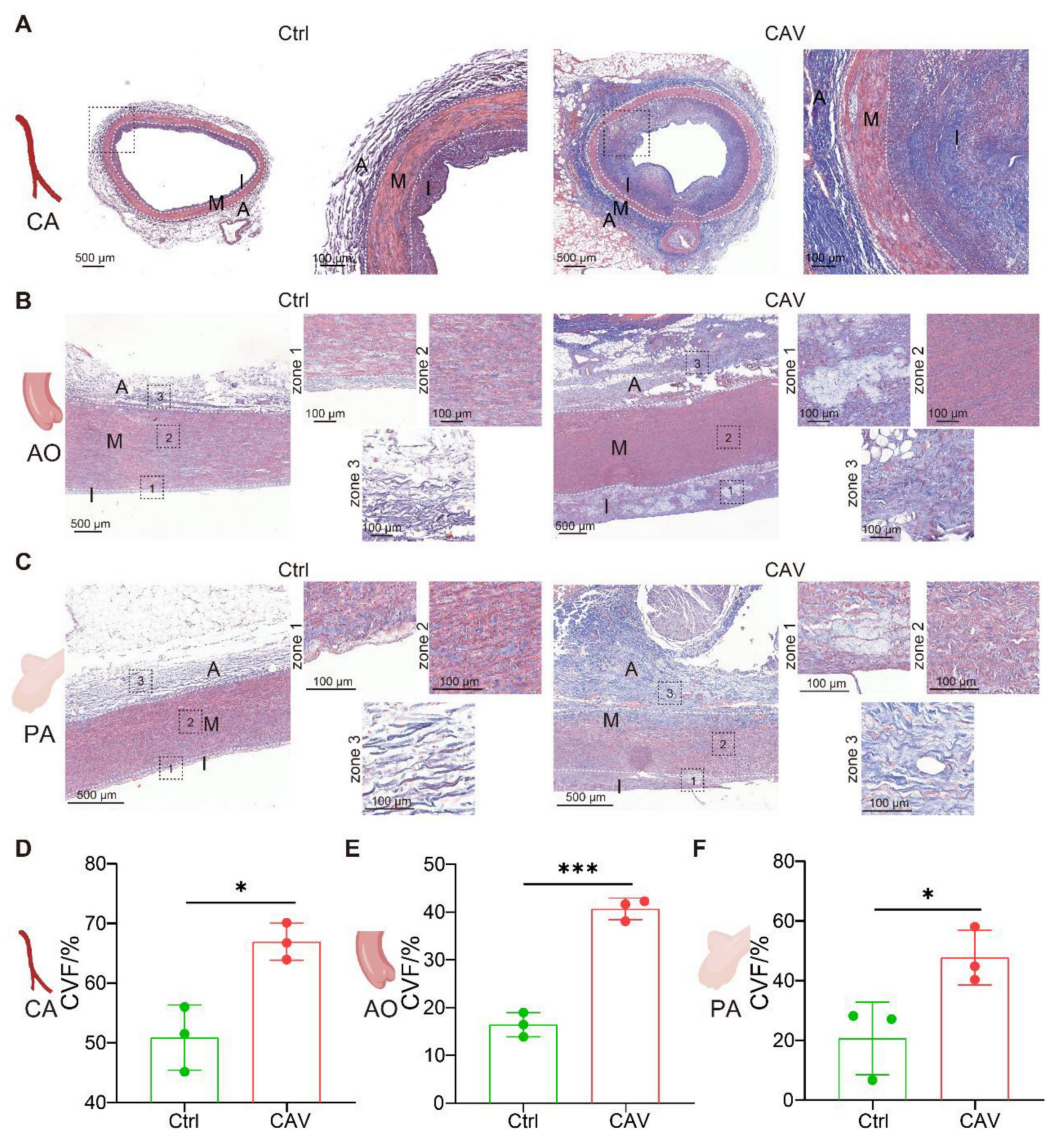
** Masson staining reveals marked fibrosis in coronary, aortic, and pulmonary arteries from CAV allografts.** (A-C) Extensive collagen deposition is observed in CAV CA, AO, and PA, predominantly in the intima and adventitia. Control vessels exhibit normal structure with minimal collagen. (D-F) Collagen volume fraction (CVF) is significantly increased in CAV samples across all three vascular beds. A: adventitia; M: media; I: intima. Data are presented as mean ± SD (n = 3). *p < 0.05, ***p < 0.001 (Student's t-test).

To assess the extent of vascular fibrosis in allografts, Masson staining was performed on CAV CA, AO, and PA (**Figure 2A-C**). Compared to controls, all three vascular beds in the CAV group exhibited extensive collagen deposition, predominantly in the intima and adventitia, indicating fibrosis-driven structural remodeling. Quantitative analysis showed a significant increase in collagen volume fraction (CVF) in CA, AO, and PA from the CAV group (**Figure 2D-F**).

These findings indicate that CAV induces not only coronary remodeling but also widespread fibrosis across large vessels, supporting pan-arterial fibrosis as a key pathological feature of CAV.

### Expanded fibroblast populations underlie pan-arterial fibrosis in CAV

**Figure 3:**
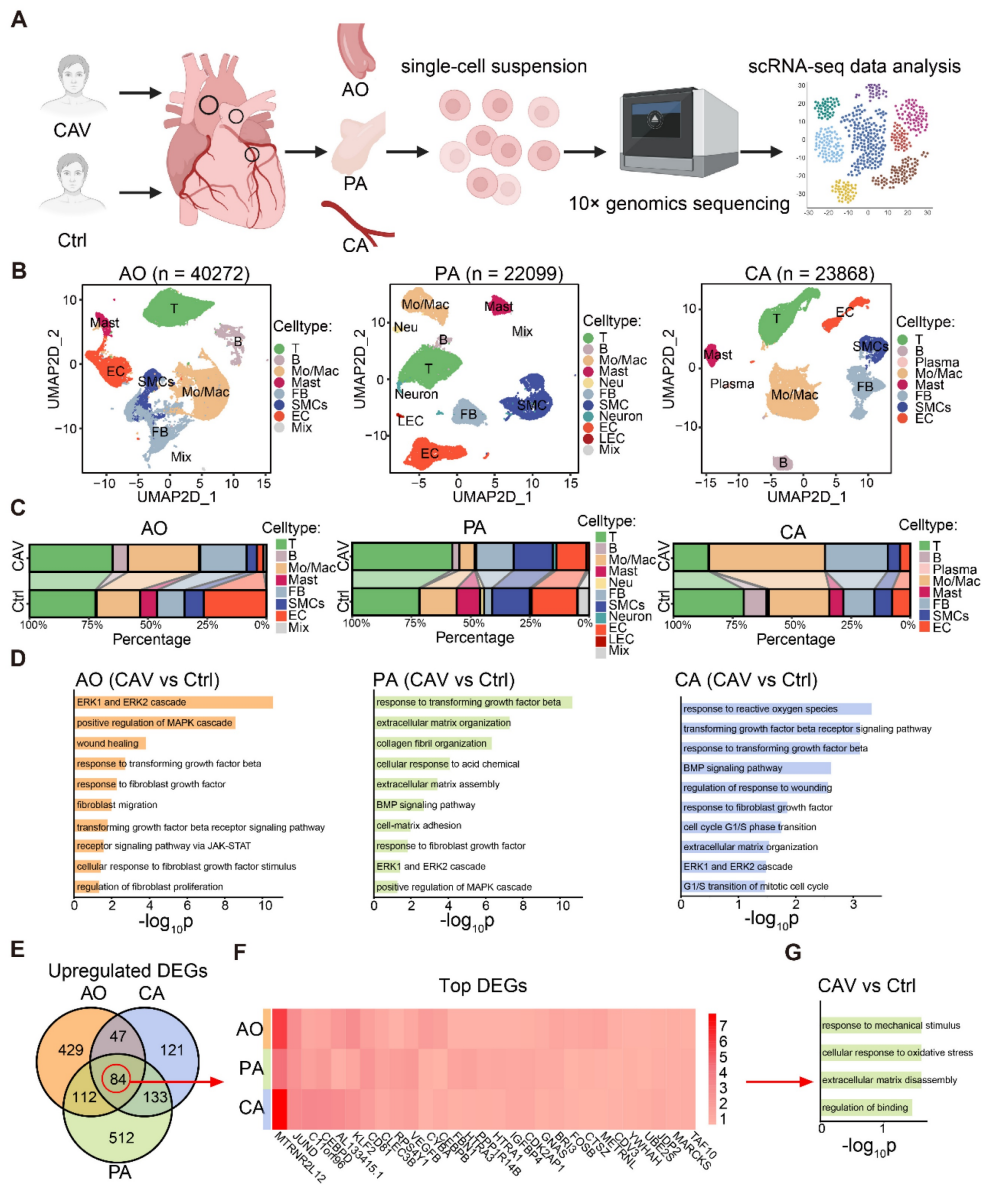
** Single-cell transcriptomic analysis reveals immune activation and fibroblast remodeling in CAV vessels.** (A) Workflow of single-cell RNA sequencing on CA, AO, and PA samples from CAV and control groups. (B) UMAP plots showing the distribution of major cell types in the three vessels. (C) Comparison of cell type proportions between CAV and control groups across all vessels. (D) GO enrichment analysis of differentially expressed FB genes, highlighting pathways related to collagen fibril formation, extracellular matrix remodeling, and fibroblast activation. (E) Venn diagram showing 84 commonly upregulated FB genes shared across CA, AO, and PA. (F) Heatmap of commonly upregulated FB genes. (G) GO enrichment results of the shared upregulated genes.

To investigate the fibrosis-related mechanisms in CAV, we performed single-cell RNA sequencing on CA, AO, and PA tissues from CAV patients (**Figure 3A**), and identified the major cell populations in each vessel (**Figure 3B and Figure S1**). The results revealed substantial differences in immune cell composition and tissue remodeling between CAV and control groups. Overall, immune cells accounted for more than half of the total cell population in both groups, with T cells and monocytes/macrophages (Mo/Mac) being the dominant subsets. In the CAV group, the proportion of immune cells was markedly increased, particularly due to the expansion of T cells and Mo/Mac, indicating heightened immune activation during chronic rejection. In CA, Mo/Mac was the predominant immune population in CAV samples, whereas T cells were more abundant in PA. AO exhibited a mixed distribution of T cells and Mo/Mac (**Figure 3B-C**).

Beyond immune changes, CAV samples showed a significant increase in FBs across all three vessels, accompanied by a marked reduction in endothelial cells (ECs) and SMCs (**Figure 3C**), consistent with fibrosis and vascular remodeling. To further explore the molecular features of FBs, we conducted GO enrichment analysis on FB-specific upregulated genes in the CAV group. Pathways such as collagen fibril organization, extracellular matrix organization, and ERK1 and ERK2 cascade were significantly enriched (**Figure 3D**). We identified 84 commonly upregulated genes in FBs from CA, AO, and PA (**Figure 3E**). Among these genes, TAF10, FOS, and JUNB indicate transcriptional activation and stress responses under chronic rejection; MARCKS and METRNL are associated with cytoskeletal remodeling and cell-matrix interactions, potentially contributing to fibroblast activation; whereas HTRA1 and complement-related genes are involved in matrix remodeling and inflammatory regulation (**Figure 3F**). Collectively, these genes exhibit consistent upregulation across the three vascular sites, suggesting a shared transcriptional program centered on inflammation-associated extracellular matrix remodeling. GO analysis further confirmed that these shared genes are enriched in extracellular matrix related pathways (**Figure 3G**). Together, these single-cell data reveal coordinated immune activation and fibroblast remodeling across multiple vessels during CAV, reinforcing pan-arterial fibrosis as a central pathological feature of this disease.

**Figure 4:**
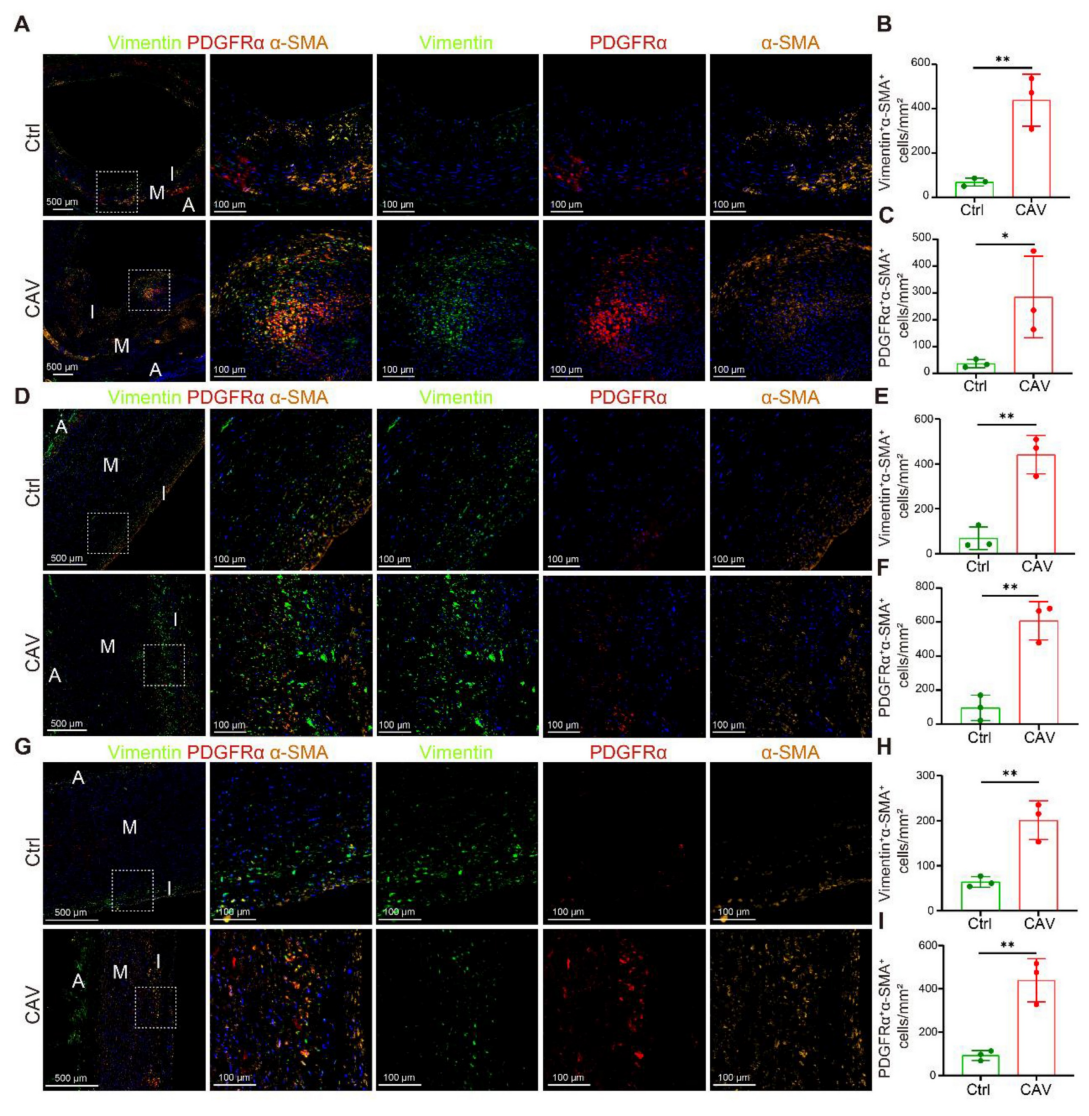
** mIHC validation of myofibroblast activation and enrichment in CAV vessels.** Abundant Vimentin⁺α-SMA⁺ and PDGFRα⁺α-SMA⁺ double-positive cells are observed in CAV CA (A-C), AO (D-F), and PA (G-I), while such cells are less frequent in controls. Myofibroblasts (myoFBs) are defined as Vimentin⁺α-SMA⁺ or PDGFRα⁺α-SMA⁺ cells, and smooth muscle cells are defined as α-SMA single-positive cells. A: adventitia; M: media; I: intima. Data are presented as mean ± SD (n = 3). *p < 0.05, **p < 0.01 (Student's t-test).

To validate the scRNA-seq findings of FB expansion, we performed mIHC on paraffin-embedded sections of CA, AO, and PA from CAV patients and controls to examine the distribution of myofibroblasts (myoFBs). MyoFBs are key effector cells in tissue fibrosis, characterized by their ability to produce extracellular matrix and contractile proteins; thus, their spatial distribution reflects the extent and pattern of fibrotic remodeling 27. Vimentin and PDGFRα were used to identify FBs. Cells co-expressing α-SMA with either Vimentin or PDGFRα were defined as myoFBs, whereas α-SMA single-positive cells were considered SMCs. In all three vessels, the CAV group exhibited abundant Vimentin⁺α-SMA⁺ and PDGFRα⁺α-SMA⁺ myoFBs, whereas such double-positive cells were rarely observed in controls (**Figure 4A**-**I**). These results are consistent with the scRNA-seq data and further support the activation and accumulation of myoFBs as a key contributor to vascular fibrosis in CAV.

### T cell-fibroblast crosstalk drives fibrotic remodeling in CAV

**Figure 5:**
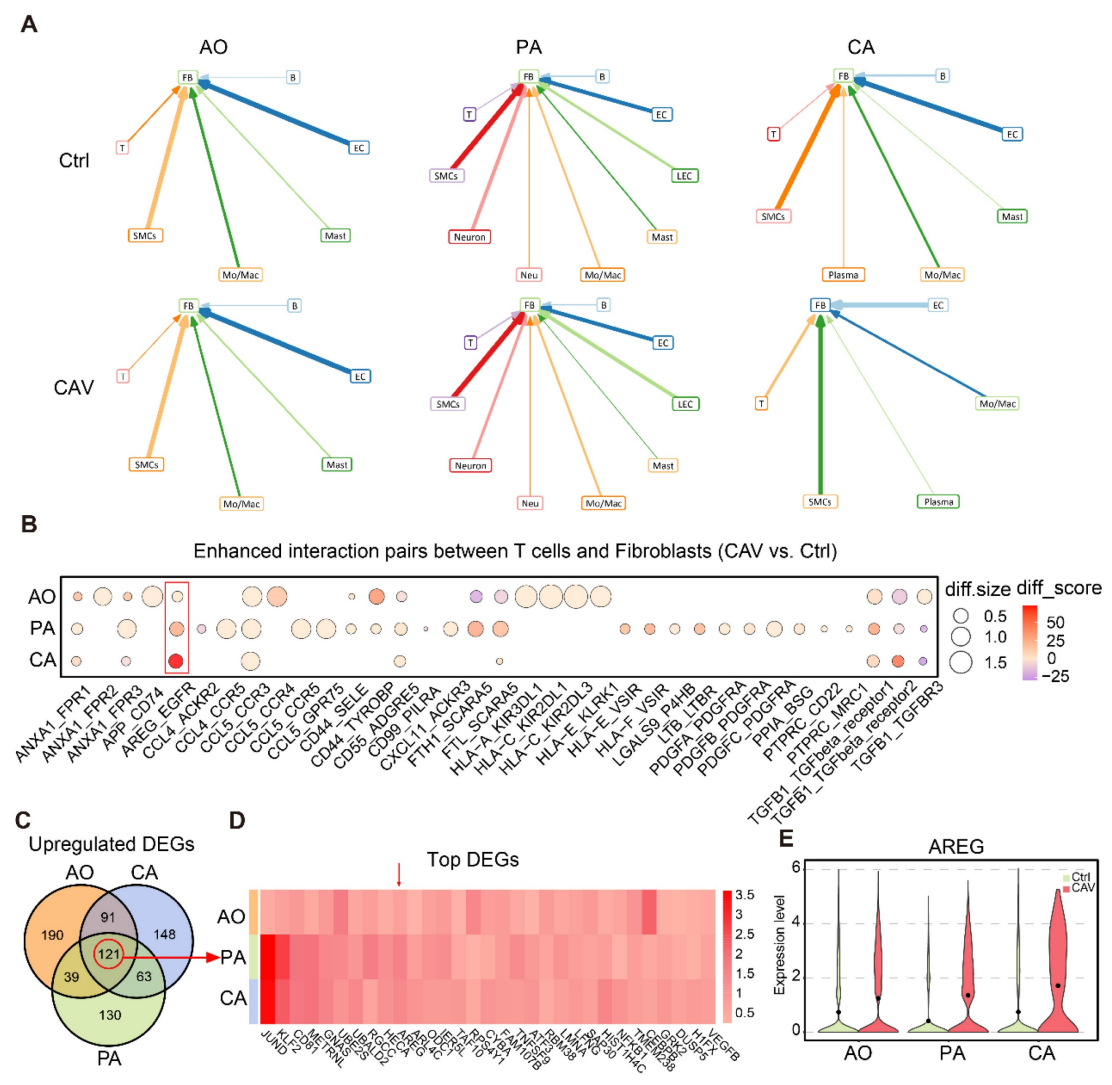
** Cell-cell interaction analysis suggests that T cells may activate FBs through AREG signaling.** (A) Cell-cell interaction analysis illustrates the interactions between different cell types and FBs in AO, PA, and CA. (B) Bubble plot of upregulated ligand-receptor pairs between T cells and FBs, with AREG-EGFR showing the most prominent increase across all three vessels. (C) Venn diagram identifying 121 commonly upregulated genes in T cells from CA, AO, and PA. (D) Heatmap of commonly upregulated genes, with AREG ranking among the top. (E) Violin plot showing significantly elevated AREG expression in T cells from the CAV group. diff.size indicates the change in interaction strength between groups, while diff.score reflects the overall reliability of the interaction.

To further explore the upstream signals driving FB expansion and activation in CAV, we performed cell-cell interaction analysis using scRNA-seq data from CA, AO, and PA. The results showed that in the CA and PA, the number of interactions between T cells and FBs was markedly higher in the CAV group than in the Ctrl group, whereas in the AO, the number of such interactions was overall comparable between the two groups (**Figure 5A and Figure S2**). Compared with other cell types, T cell-FB interactions exhibited a relatively consistent pattern across the three vascular beds, suggesting that T cells may regulate FB activation through intercellular signaling as a shared feature among the three sites. We next analyzed the specific ligand-receptor pairs contributing to the enhanced T cell-FB interactions. Several well-established pro-fibrotic signaling axes were significantly upregulated in CAV, including AREG-EGFR, TGFB1-TGFBR1/2/3, PDGFA/PDGFB/PDGFC-PDGFRA, and CD44-SELE/TYROBP (**Figure 5B**). These pathways are known to promote fibroblast activation, migration, and matrix deposition, indicating that T cells may facilitate the transition of FBs into myofibroblasts through multiple paracrine mechanisms 27-30. Notably, AREG-EGFR emerged as the most strongly upregulated and consistently enhanced interaction across all three vessels, highlighting it as a potential key signaling axis in CAV.

We further analyzed upregulated genes in T cells and identified 121 genes commonly upregulated across CA, AO, and PA (**Figure 5C**), corroborating that AREG is highly expressed at all three vessels (**Figure 5D**). *AREG* expression was significantly elevated in T cells from the CAV group compared to Ctrl group (**Figure 5E**), supporting its role as a key secreted factor contributing to FB activation.

**Figure 6:**
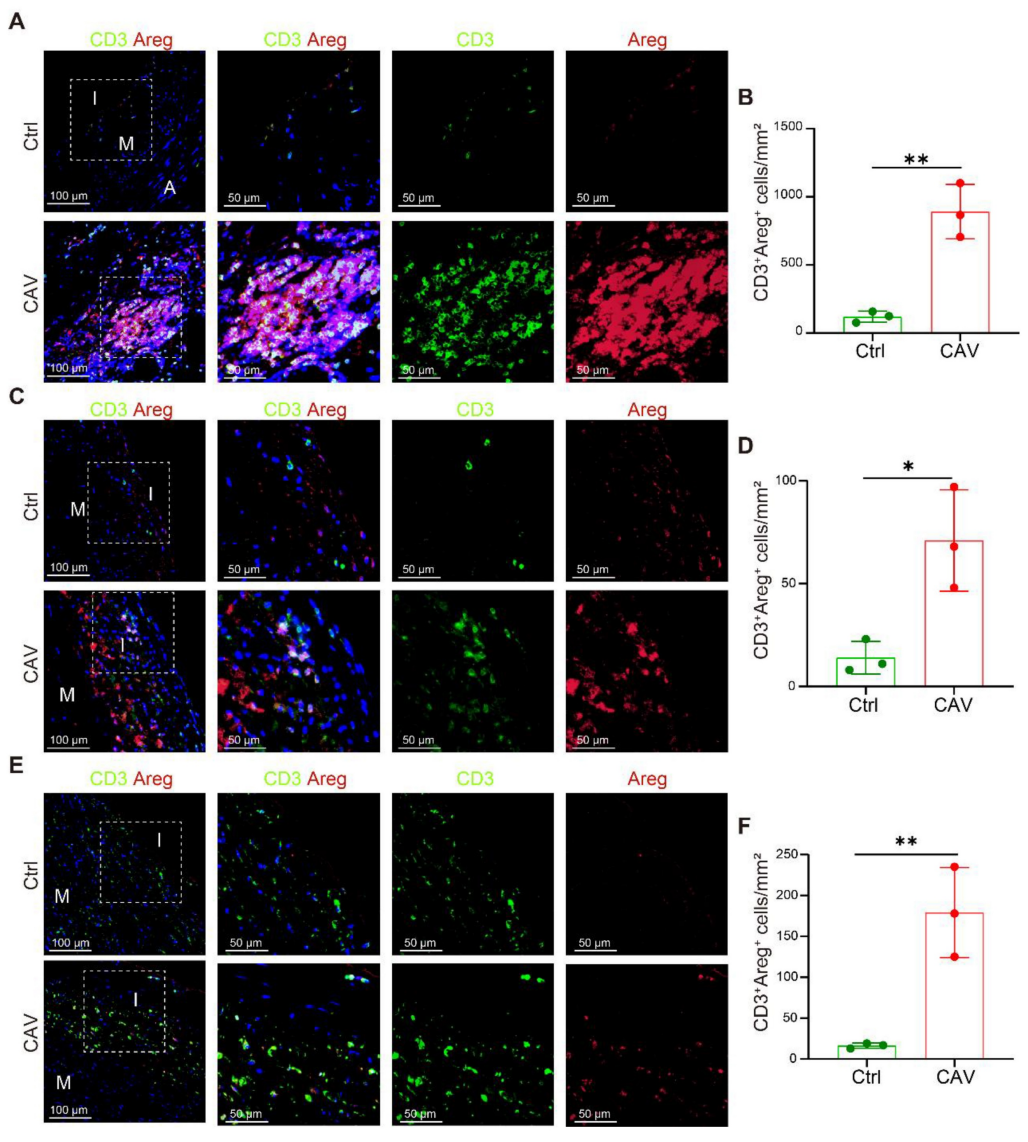
** Increased AREG expression in T cells within CAV vessels.** (A-B) CD3⁺ T cells within lymphoid cell aggregates in CAV CA show strong AREG expression. (C-F) AREG⁺CD3⁺ T cells are enriched in the vessel wall in CAV AO and PA; few positive cells are observed in controls. A: adventitia; M: media; I: intima. Data are presented as mean ± SD (n = 3). *p < 0.05, **p < 0.01 (Student's t-test).

To validate the high expression of AREG in T cells within CAV vessels, we performed dual immunostaining for CD3 and AREG on paraffin-embedded sections of CA, AO, and PA. In the CA, prominent lymphoid cell aggregates were observed in the CAV group, within which large numbers of CD3⁺ T cells exhibited high AREG expression, suggesting that these cells may continuously release AREG in the local immune microenvironment to activate FBs (**Figure 6A-B**). These aggregates predominantly consisted of mixed lymphocyte populations and were mainly located in the adventitia, which may help explain the relatively lower proportion of T cells detected by scRNA-seq, potentially due to partial loss of the adventitial layer during tissue dissociation. Although typical inflammatory cell aggregates were not observed in the AO and PA, abundant AREG⁺ T-cell infiltration was evident in the CAV group, with significantly higher numbers than in controls and a distribution mainly within the intima or the adjacent medial layer, further supporting T cells as a major source of AREG in CAV. In contrast, AREG⁺ T-cell infiltration was limited in the control group (**Figure 6D-F**).

These findings are consistent with the results of cell-cell interaction analysis, and further support the notion that T cell-derived AREG acts as a key secreted factor driving vascular fibrosis in CAV.

### Blockade of the T cell-fibroblast signaling axis alleviates graft fibrosis

**Figure 7:**
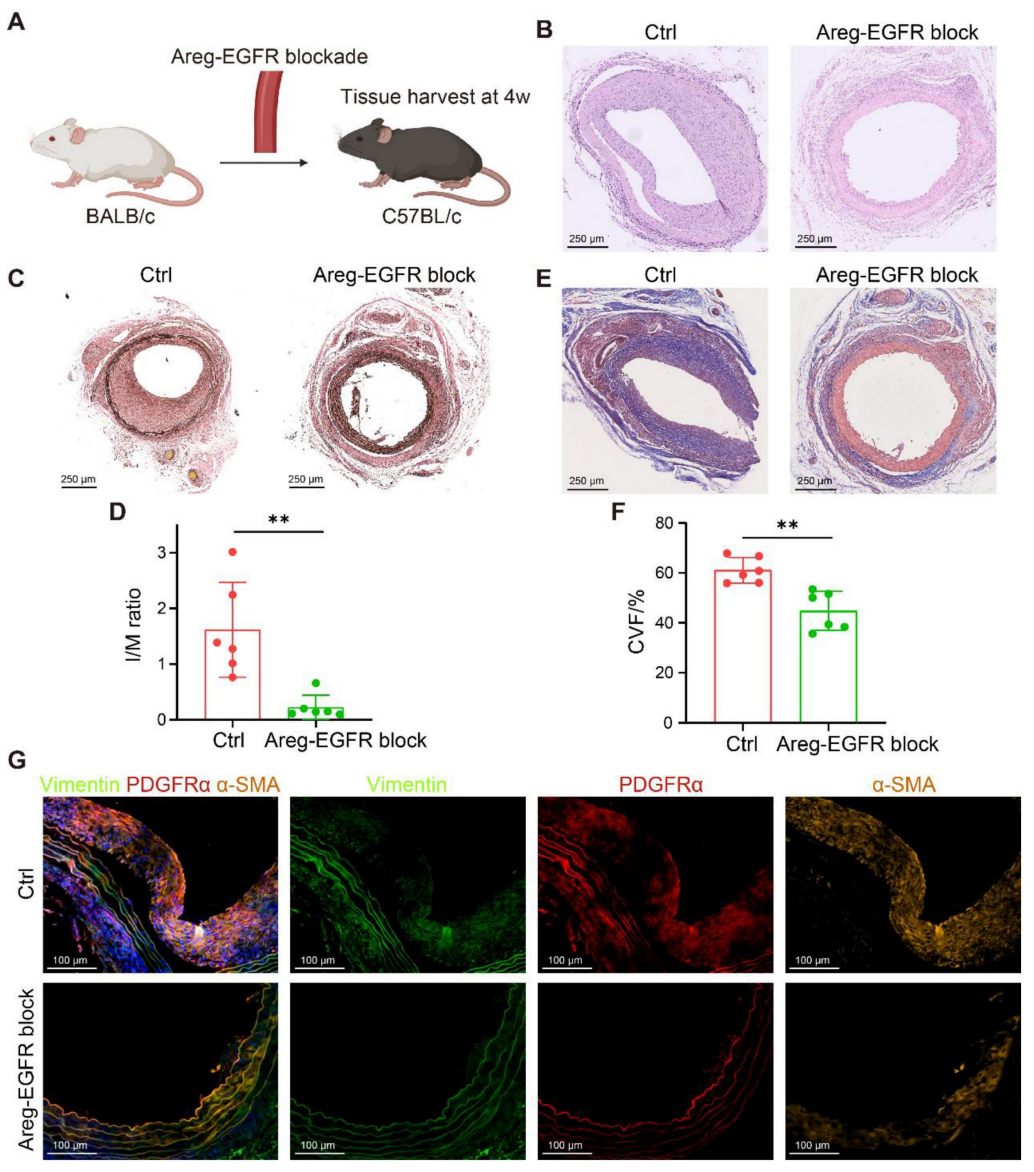
** Blockade of the AREG-EGFR signaling axis alleviates fibrosis in murine vascular grafts.** (A) Schematic of the mouse arterial transplantation model. (B) H&E staining shows that intimal hyperplasia is markedly reduced in the treatment group. (C-D) EVG staining shows that intimal hyperplasia is markedly reduced in the treatment group, with a significantly decreased intima-to-media thickness ratio (I/M ratio). (E-F) Masson staining reveals reduced collagen deposition and significantly attenuated fibrosis in the treatment group. (G) mIHC demonstrates a reduced number of Vimentin⁺α-SMA⁺ and PDGFRα⁺α-SMA⁺ myoFBs in the treated grafts. Data are presented as mean ± SD (n = 6). **p < 0.01 (Student's t-test).

To validate these findings, we established two murine transplantation models. In the arterial transplantation model, thoracic aortic segments from BALB/c mice were grafted into the carotid arteries of C57BL/6 recipients (**Figure 7A**), and the AREG-EGFR signaling axis between T cells and FBs was blocked using the EGFR inhibitor Erlotinib (50 mg/kg/day). This intervention significantly suppressed neointimal hyperplasia (**Figure 7B**) and reduced the I/M ratio (**Figure 7C-D**). Masson staining showed reduced collagen deposition and a marked attenuation of vascular fibrosis (**Figure 7E-F**). Further mIHC analysis demonstrated that the numbers of Vimentin⁺α-SMA⁺ and PDGFRα⁺α-SMA⁺ myoFBs were significantly decreased in treated grafts (**Figure 7G**), indicating a critical role of the AREG-EGFR axis in CAV-associated vascular fibrosis.

**Figure 8:**
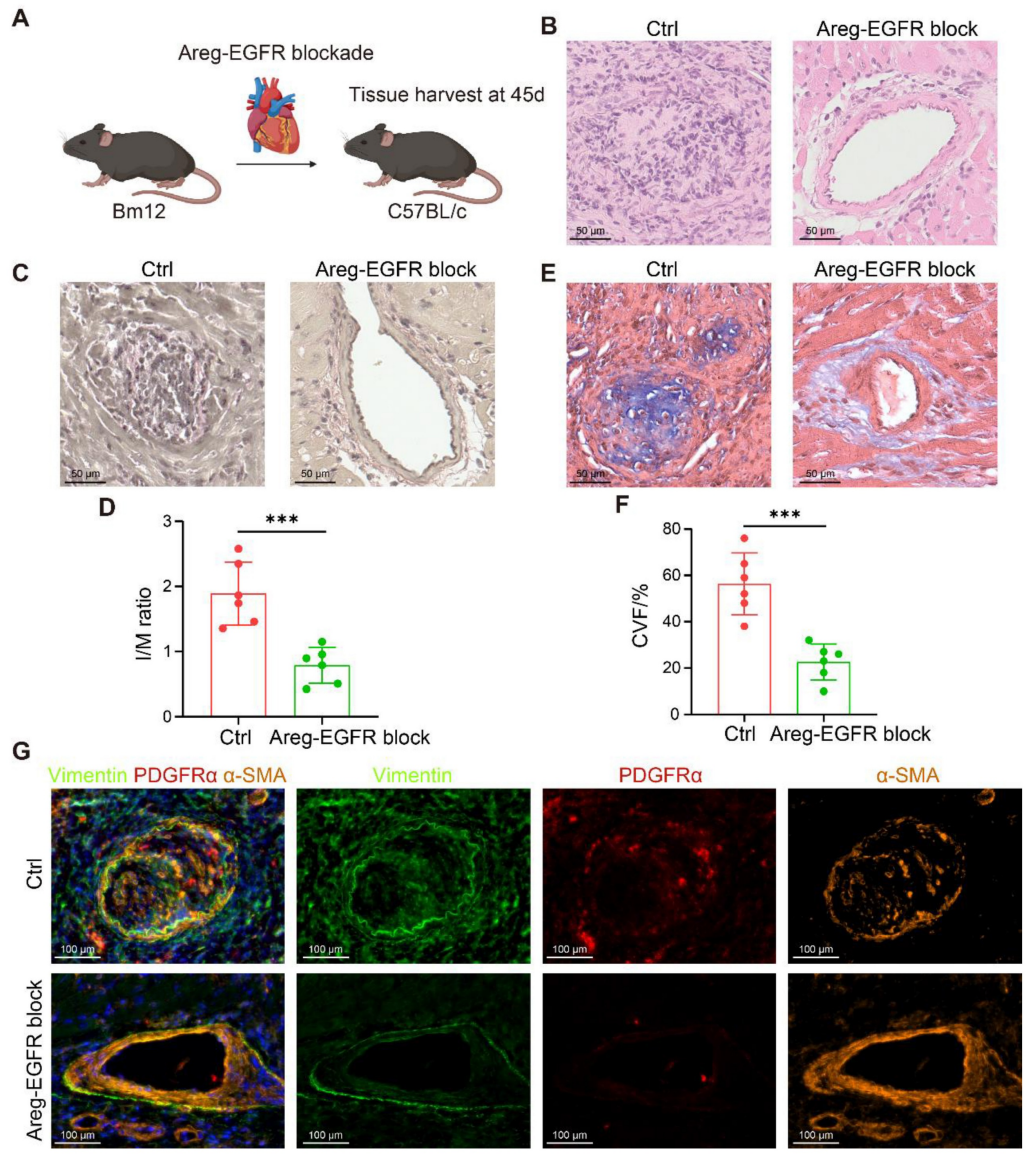
** Blockade of the AREG-EGFR signaling axis alleviates fibrosis in a murine chronic transplantation model.** (A) Schematic illustration of the bm12-to-C57BL/6 chronic transplantation model with pharmacological blockade of AREG-EGFR signaling. (B) Representative H&E staining showing that AREG-EGFR blockade markedly reduces neointimal hyperplasia. (C-D) EVG staining shows that intimal hyperplasia is markedly reduced in the treated grafts, with a significantly decreased intima-to-media thickness ratio (I/M ratio). (E-F) Masson staining reveals reduced collagen deposition and significantly attenuated fibrosis in the treated grafts. (G) mIHC demonstrates a reduced number of Vimentin⁺α-SMA⁺ and PDGFRα⁺α-SMA⁺ myoFBs in the treated grafts. Data are presented as mean ± SD (n = 6). ***p < 0.001 (Student's t-test).

To further corroborate these results, a bm12-to-C57BL/6 chronic transplantation model was established, and Erlotinib (50 mg/kg/day) was administered from postoperative day 28 to day 45 (**Figure 8A**). Blockade of the AREG-EGFR axis significantly alleviated vascular stenosis and delayed the development of CAV (**Figure 8B-D**). Masson staining further confirmed a pronounced reduction in vascular fibrosis following treatment (**Figure 8E-F**). Consistently, mIHC revealed markedly fewer Vimentin⁺α-SMA⁺ and PDGFRα⁺α-SMA⁺ myoFBs in the treated grafts (**Figure 8G**). Taken together, blockade of the AREG-EGFR pathway between T cells and FBs effectively suppresses FB activation and mitigates fibrosis, providing a potential therapeutic strategy for pan-arterial fibrosis in CAV.

## Discussion

CAV is a major barrier to long-term survival after heart transplantation, characterized by concentric narrowing of the coronary arteries 1, 2. Previous studies have suggested that intimal thickening in CA is primarily driven by myointimal hyperplasia, accompanied by atherosclerosis and inflammatory responses 31-33, which aligns with the pathological features observed in our clinical samples. Traditionally, α-SMA⁺ cells within the intima have been considered to originate mainly from SMCs; however, α-SMA can also be expressed by myoFBs. Our study further confirms that in CAV lesions, not only are SMCs enriched, but FBs are also significantly expanded, and a considerable proportion of α-SMA⁺ cells are in fact myoFBs. This finding is supported by a spatial transcriptomic study that revealed extensive vascular remodeling in CAV coronary arteries, with highly expressed transcripts largely attributed to FBs 34. Moreover, previous literature has reported that CAV plaques are rich in lipid-laden foam cells but are typically capped by thick fibrous layers and rarely rupture 35, consistent with our observations of stable atherosclerotic features.

Importantly, we extended our investigation beyond the CA to the donor heart's AO and PA, which are often overlooked in CAV research. We found that CA, AO, and PA exhibited similar pathological changes, including intimal thickening, plaque formation, and inflammatory infiltration. Further analysis demonstrated that vascular remodeling in all three vessels converged on a fibrotic outcome, forming a pattern of “pan-arterial fibrosis.” This structural remodeling markedly increased collagen deposition in the vessel wall, leading to reduced elasticity and vascular stiffness. Previous clinical studies have reported that decreased compliance of the AO and PA correlates with poor prognosis in heart transplant recipients 11, 12, and our identification of pan-arterial fibrosis provides a plausible pathological basis for this observation. While most prior studies have focused on myocardial fibrosis and its role in diastolic dysfunction 36-39, our findings emphasize that post-HTx fibrosis also profoundly affects the vascular system, significantly impacting graft function and survival, and underscoring its critical role in transplant failure.

To further investigate the mechanisms underlying vascular fibrosis, we performed scRNA-seq analysis on tissues from the three types of vessels and identified increased FBs and ECM remodeling as shared pathological features. Cell-cell interaction analysis revealed a marked enhancement of the AREG-EGFR signaling axis between T cells and FBs, suggesting it may be a key driver of FB activation. Previous studies have demonstrated that this signaling pathway plays an important role in tissue fibrosis under various pathological conditions 40-42. As a ligand for EGFR, AREG has been shown to promote fibroblast proliferation, extracellular matrix deposition, and fibrotic remodeling across multiple organ systems—findings that are highly consistent with the role of AREG-EGFR signaling in CAV-associated vascular fibrosis observed in our study. Further validation in mouse models confirmed that blockade of this pathway effectively attenuates fibrotic progression in vascular grafts. These results suggest that, in addition to standard immunosuppressive regimens, therapeutic strategies targeting pan-arterial fibrosis hold promise for chronic transplant management.

In summary, this study identifies pan-arterial fibrosis as a common pathological manifestation during CAV progression, expanding our understanding of post-HTx vascular remodeling. These findings underscore the potential of anti-fibrotic therapies as a complementary approach to improve long-term graft survival.

## Supplementary Material

Supplementary figures.

## Figures and Tables

**Table 1:** Baseline characteristics of patients

	Patient 1	Patient 2	Patient 3*
Gender	Female	Male	Male
Age at first HTx	22	24	48
Immunosuppressive regimen	Glucocorticoid, MMF, CsA	Glucocorticoid, MMF, CsA	Glucocorticoid, MMF, CsA, Sirolimus
NT-proBNP (pg/mL)**	2877	2225	1008
NYHA classification**	Class Ⅳ	Class Ⅳ	Class Ⅳ
Age at re-HTx	27	32	54
Pathology	CAV Ⅲ, ACR-2R	CAV Ⅲ, ACR-2R	CAV Ⅲ, ACR-2R

HTx, heart transplantation; re-HTx, repeat heart transplantation; CAV, cardiac allograft vasculopathy; ACR, acute cellular rejection; MMF, mycophenolate mofetil; CsA, cyclosporin A * Tissues from the aorta, pulmonary artery, and coronary artery of patient 3 were used for scRNA-seq. ** These values represent the final test results prior to re-HTx.

## Data Availability

The data supporting the findings of this study are available from the corresponding author upon reasonable request.

## Abbreviations

A: adventitia
ACR: acute cellular rejection
AO: aorta
AREG: amphiregulin
CA: coronary artery
CAV: cardiac allograft vasculopathy
CsA: cyclosporin A
Ctrl: control
CVF: collagen volume fraction
ECM: extracellular matrix
ECs: endothelial cells
EGFR: epidermal growth factor receptor
FBs: fibroblasts
GO: Gene Ontology
HTx: heart transplantation
I: intima
I/M: intima-to-media thickness ratio
M: media
MMF: mycophenolate mofetil
Mo/Mac: monocytes/macrophages
myoFBs: myofibroblasts
PA: pulmonary artery
PDGFRα: platelet-derived growth factor receptor alpha
re-HTx: repeat heart transplantation
scRNA-seq: single-cell RNA sequencing
SMCs: smooth muscle cells
α-SMA: alpha-smooth muscle actin
