# Supplementary material for: Blocking AREG-EGFR signaling attenuates pan-arterial fibrosis in chronic cardiac allograft rejection
Source: Theranostics. 2026 Feb 11;16(9):4603–18. doi: 10.7150/thno.125318 (PMC12964133; doi:10.7150/thno.125318)
Supplement: Supplementary file 1 — Supplementary figures. [file thnov16p4603s1.pdf]

Supplemental materials

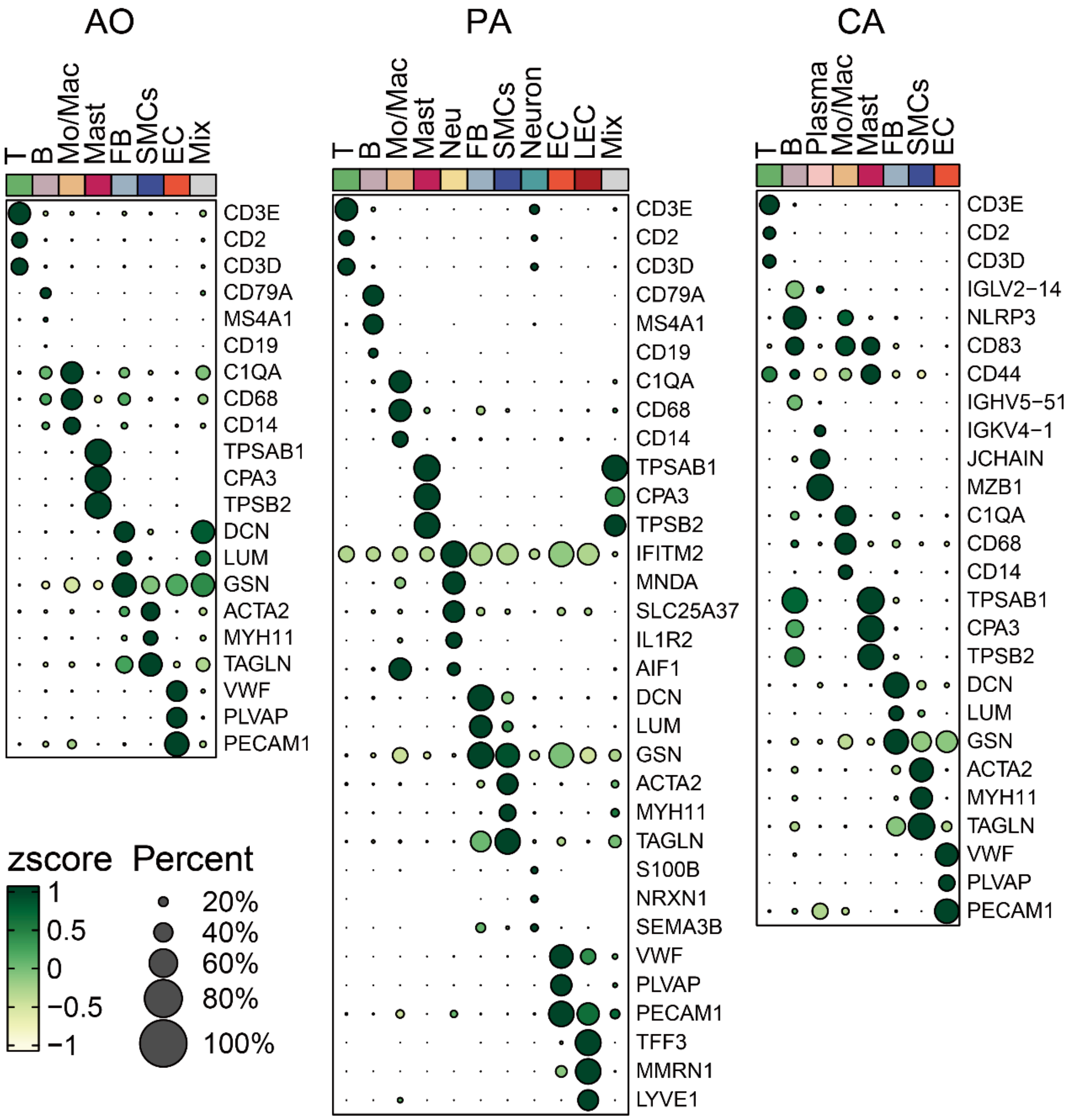

Figure S1. Marker gene expression profiles of major cell types across AO, PA, and CA. Bubble plots show representative marker gene expression across different cell populations in AO, PA, and CA.

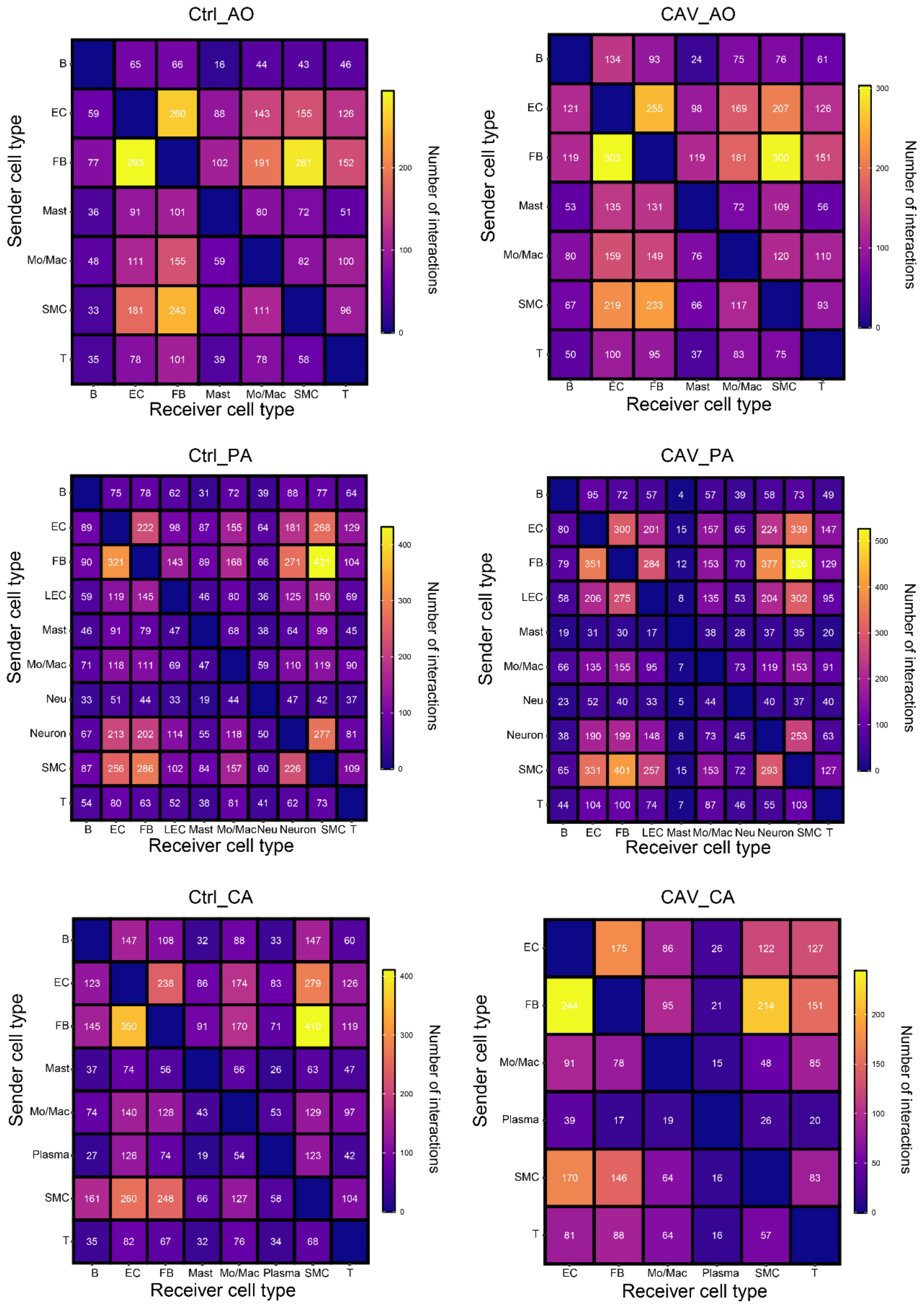

Figure S2. Cell-cell interaction heatmaps show the number of interaction pairs among different cell types in the AO, PA, and CA in the CAV and Ctrl groups.
